# Arachidonic Acid and Eicosapentaenoic Acid Metabolism in Juvenile Atlantic Salmon as Affected by Water Temperature

**DOI:** 10.1371/journal.pone.0143622

**Published:** 2015-11-24

**Authors:** Fernando Norambuena, Sofia Morais, James A. Emery, Giovanni M. Turchini

**Affiliations:** 1 School of Life and Environmental Sciences, Deakin University, PO Box 423, Warrnambool, Victoria, 3280, Australia; 2 IRTA, Ctra. Poble Nou Km 5.5, 43540, Sant Carles de la Ràpita, Spain; Northwest Fisheries Science Center, NOAA Fisheries, UNITED STATES

## Abstract

Salmons raised in aquaculture farms around the world are increasingly subjected to sub-optimal environmental conditions, such as high water temperatures during summer seasons. Aerobic scope increases and lipid metabolism changes are known plasticity responses of fish for a better acclimation to high water temperature. The present study aimed at investigating the effect of high water temperature on the regulation of fatty acid metabolism in juvenile Atlantic salmon fed different dietary ARA/EPA ratios (arachidonic acid, 20:4n-6/ eicosapentaenoic acid, 20:5n-3), with particular focus on apparent *in vivo* enzyme activities and gene expression of lipid metabolism pathways. Three experimental diets were formulated to be identical, except for the ratio EPA/ARA, and fed to triplicate groups of Atlantic salmon (*Salmo salar*) kept either at 10°C or 20°C. Results showed that fatty acid metabolic utilisation, and likely also their dietary requirements for optimal performance, can be affected by changes in their relative levels and by environmental temperature in Atlantic salmon. Thus, the increase in temperature, independently from dietary treatment, had a significant effect on the β-oxidation of a fatty acid including EPA, as observed by the apparent *in vivo* enzyme activity and mRNA expression of *pparα* -transcription factor in lipid metabolism, including β-oxidation genes- and *cpt1* -key enzyme responsible for the movement of LC-PUFA from the cytosol into the mitochondria for β-oxidation-, were both increased at the higher water temperature. An interesting interaction was observed in the transcription and *in vivo* enzyme activity of *Δ5fad*–time-limiting enzyme in the biosynthesis pathway of EPA and ARA. Such, at lower temperature, the highest mRNA expression and enzyme activity was recorded in fish with limited supply of dietary EPA, whereas at higher temperature these were recorded in fish with limited ARA supply. In consideration that fish at higher water temperature recorded a significantly increased feed intake, these results clearly suggested that at high, sub-optimal water temperature, fish metabolism attempted to increment its overall ARA status -the most bioactive LC-PUFA participating in the inflammatory response- by modulating the metabolic fate of dietary ARA (expressed as % of net intake), reducing its β-oxidation and favouring synthesis and deposition. This correlates also with results from other recent studies showing that both immune- and stress- responses in fish are up regulated in fish held at high temperatures. This is a novel and fundamental information that warrants industry and scientific attention, in consideration of the imminent increase in water temperatures, continuous expansion of aquaculture operations, resources utilisation in aquafeed and much needed seasonal/adaptive nutritional strategies.

## Introduction

As global sea temperatures rise and market demands grow, aquaculture is increasingly encountering sub-optimal conditions due to changing environments [1] and/or expansion into areas outside the ideal range for the species; and this is the case for Atlantic salmon farming along the Australian coast [2]. A common acclimation of fish to altered temperature is the restructuring of the membrane lipids (homeoviscous adaptation), which is the only structural element of the cells that can be reshaped as the environmental temperature changes [3]. Therefore, following changes in water temperature, the composition of lipid membranes is altered in order to avoid damage and possibly even death, which could occur if this acclimation does not happen quickly enough [4–6]. Long chain polyunsaturated fatty acids (LC-PUFA) have unique roles in controlling and regulating cell membrane fluidity, and participate in a wide range of physiological processes including immune and stress response, growth, survival and performance [7, 8]. Hence, LC-PUFA and their derivative compounds are involved in a network of pathways which are among the most complex in live organisms, including fish [9, 10].

Most marine fish species, which lack the ability to synthesize LC-PUFA from their 18-carbon precursor fatty acids such as α-linolenic acid (18:3n-3) and linoleic acid (18:2n-6), require preformed dietary LC-PUFA, such as docosahexaenoic acid (DHA, 22:6n-3), eicosapentaenoic acid (EPA, 20:5n-3) and arachidonic acid (ARA, 20:4n-6), for their normal growth and development [11]. On the other hand, it is generally accepted that freshwater fish and anadromous salmonids can convert C_18_ PUFA of both the n-6 and n-3 series to their longer chain LC-PUFA [12], even if the final steps in the synthesis are relatively inefficient and therefore LC-PUFA requirement cannot be met without some direct dietary supply [13]. It is known that fish subjected to low water temperature increase membrane fluidity by enhancing LC-PUFA levels, and selected desaturation reactions, particularly those involved in the production of LC-PUFA, proceed more rapidly in cold than in warmer water [14]. On the other hand, numerous studies on several teleost species have shown that increase in temperature reduces the accumulation of LC-PUFA in body lipid depots and cell membranes and enhances the deposition of saturated fatty acids, SFA [6, 15–18]. Furthermore, several physiological processes are directly affected by water temperature in fish, from digestive physiology [19–21] to lipid metabolism [14]. For instance, water temperature exerts a major impact in fatty acid metabolism and use of energy depots in teleost fish [22], and therefore rises in temperature may change the fish’s requirements for optimal performance of dietary lipids and LC-PUFA [3, 5], as well as energy demand, associated to increases in growth rate. As such, during periods of high growth rate, fish β-oxidize even essential fatty acid when given in surplus [23]. An association between water temperature and fatty acid β-oxidation has been shown, but with inconsistent results. A study of fish held under simulated natural temperature indicated that β-oxidation of SFA in muscle increase during spring [24], while in vitro studies of hepatocytes suggested that β-oxidation was higher at 5°C compared with 12°C [25]. Studies looking at the swimming rate of rainbow trout at different temperatures (5°C vs 15°C) showed that with increases in temperature the critical swimming speed increases up to 52% and that lipids remain the most important fuel in these conditions [26], supporting the concept that lipid are the major energy source used by fish during aerobic exercise. Studies of fatty acid composition and swimming performance in Atlantic salmon held at 8°C suggest that the energy demanded by fish swimming is mainly provided by β-oxidation of 18-carbon unsaturated fatty acids [27], but this might change with increases in water temperature. Recent studies on fish acclimation to high temperatures, showed that aerobic scope increase together with lipid metabolism as a fish plasticity response for a better acclimation to high water temperature [28].

Dietary fat has gained much prominence for the role of LC-PUFA in regulating gene expression of many genes involved in fatty acid and glucose metabolism in mammals [29, 30]. These FA affect gene expression through various mechanism including changes in membrane composition, intracellular calcium level and eicosanoid production [31]. Furthermore, LC-PUFA and their metabolites are ligands for peroxisome proliferator–activated receptors (*ppars*) together with liver X receptor (*lxr*), partner retinoic X receptor (*rxr*), and thereby affect the expression of many lipid metabolism genes [32–34]. As such, studies in rats has shown that dietary EPA is a responsible for the hypotriglyceridemic effect of fish oil, decreasing availability of fatty acids for triacylglycerol synthesis by increasing mitochondrial β-oxidation and decreasing triacylglycerol formation caused by inhibition of diacylglycerol acyltransferase [35, 36]. Similar effects of fish oil in FA biosynthesis and β-oxidation have also been found in fish [37, 38]. Further, recent studies in teleost have reported that ARA down-regulates the expression of genes involved in lipogenesis but also in mitochondrial lipid β-oxidative related genes like carnitine acyltransferase I (*cpt1*), and the transcriptional factor *pparα* [39]; and accordingly, other two recent studies showed that the transcription rate of these genes were reduced with increase of dietary ARA in fish [40, 41]. From a fatty acid bioconversion (anabolic) point of view, dietary ARA has been reported to affect the expression of elongase (*elovl5a*) and desaturase (*Δ4fad*) genes in male broodstock fish, particularly during the reproductive season [42]. In mammals, *in vitro* studies performed in mouse lymphoma showed that ARA regulates unsaturated fatty acid biosynthesis, by inhibiting steraoyl-CoA 9-desaturase (*Δ9fad*) gene expression [43]. In suckling pigs, it has been shown that low ARA levels in the diet up-regulate *Δ6fad* expression during earlier development, indicating an effect of dietary ARA in modulating PUFA biosynthesis, which in turn should be regulated by physiological requirements, including the synthesis of eicosanoids [44].

In modern salmonid aquaculture, shortages in marine-derived oils have forced the feed industry to include elevated concentrations of alternative terrestrial oils, resulting in a concomitant reduction of LC-PUFA and bioactive lipids like ARA, EPA and DHA. Therefore, several studies have focused on the biological effects of n-3 LC-PUFA, primarily how EPA and DHA function in a range of marine and freshwater fish species and also on the optimal dietary levels to support growth of fish fed diets with fish oil replaced by vegetable oils [45–48]. In fish, ARA is mainly stored in polar lipids and is a minor component of cell membranes compared to EPA [49, 50]. Nevertheless, it is the most prominent n-6 LC-PUFA from a functional standpoint associated with membrane phospholipids, being released by the action of cytosolic phospholipase (*cpla2*), and then metabolized by cyclooxygenase (*cox-2*) and lipoxygenase (*5-lox*) into highly bioactive eicosanoids, which are involved in tissue homeostasis, inflammation and cardiovascular responses [51, 52]. However, although ARA is considered one of the most biologically relevant LC-PUFA in mammals, studies focusing on n-6 fatty acids (FA), including ARA, in fish nutrition have been somewhat neglected due to the n-3 LC-PUFA-centred research effort. More recently, some studies have focused on evaluating the importance of ARA in fish nutrition, with gradual elucidation of the physiological functions of their bioactive eicosanoid derivatives, and effects on fish performance and fatty acid digestibility [19, 40, 53]. In addition to defining optimal dietary requirements for ARA and the resultant effects on fish performance, such studies have shown that ARA and its metabolites exert complex control over many biological systems, including cardiovascular, endocrine and immune systems [54–57]. However, studies on the effects of dietary EPA and ARA are often complicated by the fact that both not only compete for incorporation into membrane phospholipids but also for biosynthesis from their respective n-3 and n-6 fatty precursors, and share the same enzyme in their metabolisation and eicosanoid production [31, 58].

These observations suggest that given the influence and competition of ARA and EPA over several metabolic systems, their dietary provision likely plays an important role in fish plasticity when adapting to sub-optimal high water temperatures. Cultured fish are fed high fat diets containing finite marine sources of LC-PUFA and, resulting from seasonal variations, are often subjected to gradual modification of water temperature that might modulate their dietary requirements. Therefore, a better understanding of ARA and EPA interactions and influence over lipid metabolism is required in order optimize the essential FA that can be provided by diets and maximize the energy that can be supplied by dietary lipids under these conditions. As such, the present study aimed at investigating the effect of high water temperature on the regulation of fatty acid metabolism in juvenile Atlantic salmon (*Salmo salar*) fed different dietary ARA/EPA ratios, with a particular focus on apparent *in vivo* anabolic and catabolic enzyme activities and expression of genes involved in lipid metabolism, more specifically in LC-PUFA biosynthesis (fatty acyl elongases and desaturases), lipogenesis (fatty acid synthase-*fas*), β-oxidation (carnitine palmitoyl transferase 1-*cpt1* and acyl-CoA oxidase-*aco*) and LC-PUFA mobilization from biomembranes (c*pla2*), as well as their regulation (several peroxisome proliferator-activated receptor-*ppar* and sterol regulatory element binding protein 1-*srebp1*).

## Materials and Methods

### Ethics Statement

All animals and procedures used in this experimentation were approved by the Deakin University Animal Welfare Committee (Number B06-2013). All possible steps towards minimising animal suffering were taken.

### Experimental diets

The present study reports a set of new data originating from an *in vivo* trial that was object of previously published studies and detailed methodological information can also be found in Trullàs *et al*. (2015) and Norambuena *et al*. (2016). The latter studies focused on the effects of altered dietary n-3/n-6 LC-PUFA ratio on nutrients and FA digestibility, and on the effects on fish performance and tissues’ fatty acid composition, respectively. The present study reports on dietary ARA/EPA metabolism of juvenile Atlantic salmon, focusing on anabolic and catabolic enzyme activities and mRNA levels of lipid metabolism genes during the second part of the *in vivo* trial, when fish were housed at two different water temperatures.

Briefly, three iso-proteic, iso-lipidic and iso-energetic diets were specifically formulated and manufactured, varying only in their fatty acid composition in terms of ARA/EPA ratio, via modification of the added dietary lipid sources. Therefore, three specifically formulated oil blends were developed, using four readily available plant based oils (canola/rapeseed, linseed, sunflower and palm oil) and three specialty (refined/concentrated) oils, each with a high content of DHA, EPA and ARA, respectively. The blends of these oils were specifically designed towards achieving three final experimental diets characterised by having: i) the same total content of saturated fatty acid (SFA), total monosaturated fatty acid (MUFA), polyunsaturated fatty acid (PUFA), LC-PUFA, n-3 C18PUFA, n-6 C18PUFA and DHA; ii) the same total content of EPA + ARA; and iii) three different EPA/ARA ratios. The experimental diets were accordingly named D-ARA (ARA/EPA ratio = 2.4), D-ARA/EPA (ARA/EPA ratio = 0.7) and D-EPA (ARA/EPA ratio = 0.1). The fatty acid composition of the three experimental diets is reported in Table 1. The manufacturing methods of the experimental diets have been described previously in detail in Trullàs *et al*. (2015) and Norambuena *et al*. (2016).

**Table 1:** Total fatty acid (FA) and fatty acid composition (mg/g lipid) of the three experimental diets.

	Experimental diets ^1^		
	D-ARA	D-ARA/EPA	D-EPA
*FA* ^*2*^ *(mg/g lipid)*			
Total FA	746.05	737.09	734.29
Total SFA^3^	159.64	157	155.01
18:1n-9	282.07	276.21	268.78
Total MUFA^4^	319.24	314.11	308.25
18:2n-6	86.26	84.31	85.02
20:4n-6	49.19	26.92	7.05
22:4n-6	0.41	0.3	0.22
22:5n-6	1.07	0.79	0.66
Total n-6 PUFA^5^	148.06	121.25	99.52
18:3n-3	74.07	77.46	78.26
20:5n-3	20.87	40.93	62.27
22:5n-3	1.54	1.9	2.37
22:6n-3	17.7	16.53	17.33
Total n-3 PUFA^6^	119.1	144.74	171.49
Total PUFA^7^	267.16	265.98	271.02
Total n-6 LC-PUFA^8^	55.91	31.21	8.81
Total n-3 LC-PUFA^9^	41.77	60.95	83.83
Total LC PUFA^10^	97.68	82.16	92.64
*FA ratios*			
ARA/EPA ^11^	2.36	0.66	0.11
LC-PUFA n-3/n-6^12^	0.75	1.95	9.52

^1^ Experimental diets abbreviations: details relative to experimental diets formulation and raw materials are avilable at Trullàs *et al*. (2015).

^2^ FA = fatty acids.

^3^ SFA = saturated fatty acids.

^4^ MUFA = monounsaturated fatty acids.

^5^ n-6 PUFA = omega-6 polyunsaturated fatty acids.

^6^ n-3 PUFA = omega-3 polyunsaturated fatty acids.

^7^ PUFA = polyunsaturated fatty acids.

^8^ n-6 LC-PUFA = long chain omega-6 polyunsaturated fatty acids.

^9^ n-3 LC-PUFA = long chain omega-3 polyunsaturated fatty acids.

^10^ Total long chain polyunsaturated fatty acid

^11^ARA/EPA = ratio of 20:4n-6/20:5n-3

^12^ LC-PUFA n-3/n-6 = ratio of long chain n-3 PUFA/n-6 PUFA.

### Fish husbandry

Juvenile Atlantic salmon (~55 g) were sourced from a private aquaculture farm (Mountain Fresh Trout and Salmon Farm, Harrietville, Australia). After transportation to the Deakin University Aquaculture Research Facility (Warrnambool, Victoria, Australia), fish were acclimatised to the new environmental conditions for a two weeks period, during which they were fed a commercial diet (Ridley Aquafeed, Narangba, Queensland, Australia). Two identical fresh water multiple-tank (1,000 L), thermostatically controlled, recirculating aquaculture systems, equipped with physical and biological filtration and UV sanitation, were used for the *in vivo* trial. Both systems were maintained on a 12:12 h light:dark cycle and with a flow rate of 10 L/min per tank; and water quality parameters were maintained at optimal levels for Atlantic salmon. Five hundred and forty fish were weighed and initially stocked in one system, with water temperature set at 10°C, and randomly distributed into 9 tanks (60 fish per tank). Tanks were randomly allocated to one of the three experimental diets in triplicate. Fish were fed twice daily to apparent satiation at 0900 and 1600 hrs. After 14 weeks, all fish from each tank were weighed, 6 fish per tank were sampled (3 fish per tank for whole body analyses and 3 fish per tank for molecular analysis) and the remaining fish were split into two groups of 20 fish each, of which one was moved to the second system, and the other was returned to their previous tank. This was considered time zero, and the commencement of the trial in the present study. After fish transfer, the water temperature of the second system was gradually increased at a rate of 1.5°C/day, from 10°C to 20°C, whereas the first system was maintained at 10°C. After an additional 6 weeks from the splitting of the fish into the two systems at different temperatures (time zero), all fish were weighed and a further 6 fish per tank were sampled (3 fish per tank for whole body analyses and 3 fish per tank for molecular analyses), representing the end of the trial.

### Chemical analyses and fatty acid metabolism assessment

The chemical composition of the experimental diets and whole body fish were determined via proximate composition analysis according to standard methods [59]. Lipid content was determined by dichloromethane:methanol extraction (2:1) technique [60], with the substitution of chloroform with dichloromethane for safety reasons and the addition of butylated hydroxytoluene (BHT) (50 mg L-1) to reduce lipid oxidation during processing. After lipid extraction, an aliquot was used for fatty acid analysis, which was implemented via trans-methylation and gas chromatography, following the procedures previously described in detail [61].

Evaluation of the *in vivo* fatty acid metabolism (apparent *in vivo* β-oxidation, bioconversion and deposition) was performed using the whole-body fatty acid balance method, as initially proposed and described [62], with further development [63]. Data relative to growth performance and feed intake, dietary and tissues fatty acid composition, and fatty acid digestibility, which were previously reported and discussed [19, 64], were used in the computations required for the implementation of the method. For reference, the initial and final body weights, the total dietary intake and the fatty acid composition of fish whole bodies resulting from the dietary intervention is reported in Table 2, and in this study should be considered as part of the methodology, rather than results, as this dataset was used for the computation of the fatty acid metabolism. Final results of the whole-body fatty acid balance method were then reported as apparent *in vivo* enzyme activity, expressed as nmol/g/day, and also as metabolic fate of dietary FA (as β-oxidation, bioconversion or deposition), expressed as % of net intake.

**Table 2:** Initial and final body weights, feed intake, total fatty acids (mg/g lipid) and fatty acid composition (mg/g lipid) of whole body of Atlantic salmon fed experimental diets with different ARA/EPA ratios and reared at two different water temperatures.

	10°C			20°C			P value^1^		
	D-ARA	D-ARA/EPA	D-EPA	D-ARA	D-ARA/EPA	D-EPA	Diet	Temp.	Inter.
Initial weight (g)	162.1±4.66	162.5±4.57	161.0±2.15	162.1±4.83	162.6±1.65	163.6±3.74	*n*.*s*.	*n*.*s*.	*n*.*s*.
Final weight (g)	212.8±5.58b	245.7±8.59ab	242.9±6.29ab	228.0±9.53b	273.1±6.99a	260.1±8.73ab	***	***	*n*.*s*.
Feed intake (g/day)	1.2±0.08c	1.3±0.02c	1.4±0.08c	1.8±0.08b	2.3±0.09a	2.0±0.03ab	***	***	*n*.*s*.
*TFA (mg/g lipid)* ^*2*^	771.1±8.93	775.3±6.05	773.8±4.72	780.7±14.50	785.6±4.82	777.6±10.27	*n*.*s*.	*n*.*s*.	*n*.*s*.
Fatty Acids (mg g)								
14:0	10.2±0.25ab	10.1±0.17ab	10.6±0.13a	9.5±0.24b	9.8±0.25	9.6±0.14b	*n*.*s*.	***	*n*.*s*.
16:0	92.4±1.44c	95.8±0.95abc	99.7±1.07ab	94.3±2.10bc	98.9±1.22	101.1±1.51a	***	*n*.*s*.	*n*.*s*.
18:0	37.4±0.64abc	35.9±0.68bc	34.2±0.31c	39.9±1.12a	38.0±0.40b	34.7±0.89c	***	**	*n*.*s*.
Total SFA^3^	146.4±2.32	148.5±1.69	150.8±1.51	150.5±3.78	153.7±1.70	151.7±2.62	*n*.*s*.	*n*.*s*	*n*.*s*.
16:1n-7	19.3±0.50ab	19.4±0.21ab	19.9±0.26a	17.7±0.38c	18.6±0.35ab	18.2±0.36bc	*n*.*s*.	***	*n*.*s*.
18:1n-7	16.0±0.21	16.0±0.13	16.4±0.15	15.5±0.29	15.8±0.16	15.8±0.21	*n*.*s*.	*n*.*s*.	*n*.*s*.
18:1n-9	274.4±3.05	275.8±1.79	272.9±1.88	279.2±4.65	279.8±1.72	272.3±3.19	*n*.*s*.	*n*.*s*.	*n*.*s*.
20:1n-9	9.5±0.26a	8.5±0.13b	8.3±0.16b	8.7±0.18b	8.4±0.09	7.6±0.07c	***	***	*n*.*s*.
24:1n-9	1.7±0.05	1.6±0.05	1.6±0.04	1.6±0.05	1.7±0.02b	1.6±0.03	*n*.*s*.	*n*.*s*.	*n*.*s*.
TOTAL MUFA^4^	326.2±3.78	326.1±2.08	324.0±2.36	327.9±5.65	329.4±2.18	320.3±3.83	*n*.*s*.	*n*.*s*.	*n*.*s*.
18:2n-6	80.6±1.00	81.4±0.66	81.0±0.53	83.7±1.42	83.6±0.48	83.2±1.02	*n*.*s*.	**	*n*.*s*.
20:2n-6	5.1±0.13a	3.8±0.61ab	3.9±0.08ab	4.8±0.11ab	4.3±0.13b	3.4±0.57b	***	*n*.*s*.	*n*.*s*.
20:3n-6	7.0±0.08a	5.0±0.06b	3.4±0.06c	6.9±0.14a	4.9±0.06ab	3.2±0.06c	***	*n*.*s*.	*n*.*s*.
20:4n-6	41.4±0.32b	23.4±0.33b	7.0±0.10d	43.7±0.87a	24.3±0.40c	8.5±0.31e	***	*n*.*s*.	*n*.*s*.
22:4n-6	5.1±0.25a	2.7±0.07b	1.0±0.02c	5.5±0.39a	2.8±0.08b	1.1±0.03c	***	*n*.*s*.	*n*.*s*.
22:5n-6	5.4±0.14a	3.0±0.12b	1.4±0.08c	5.5±0.19a	3.5±0.07b	1.9±0.19c	***	**	*n*.*s*.
Total n-6 PUFA^5^	149.3±1.45a	124.0±0.88b	101.3±0.67c	154.4±2.62a	126.1±0.95	104.0±1.45c	***	**	*n*.*s*.
18:3n-3	50.2±0.57d	54.7±0.52bc	55.6±0.24b	52.9±0.77cd	57.0±0.73c	58.7±0.78a	***	***	*n*.*s*.
20:3n-3	0.5±0.06c	3.2±0.76b	4.3±0.10ab	0.5±0.05c	4.8±0.12c	4.4±0.18ab	***	*n*.*s*.	*n*.*s*.
20:4n-3	5.2±0.09cd	6.2±0.11ab	6.7±0.07a	4.7±0.11d	5.6±0.11	6.4±0.26a	***	***	*n*.*s*.
20:5n-3	16.5±0.30c	27.4±0.37b	38.9±0.66a	15.9±0.42c	26.4±0.29ab	37.9±0.37a	***	*n*.*s*.	*n*.*s*.
22:5n-3	9.1±0.07c	11.5±0.30b	14.4±0.29a	7.7±0.49c	11.3±0.50	13.8±0.37a	***	**	*n*.*s*.
22:6n-3	49.1±0.96bc	51.6±0.51ab	54.2±0.38a	47.8±0.93c	50.0±0.98b	54.4±0.98a	***	*n*.*s*.	*n*.*s*.
Total n-3 PUFA^6^	146.1±2.14c	173.0±2.23b	192.9±1.01a	145.3±2.78c	172.7±2.40b	197.0±3.02a	***	*n*.*s*.	*n*.*s*.
Total PUFA^7^	295.4±3.45	297.0±2.56	294.2±1.49	299.7±5.30	298.8±3.16b	301.0±4.41	*n*.*s*.	*n*.*s*.	*n*.*s*.
Total n-6 LC PUFA^8^	64.4±0.50a	38.8±0.57b	17.7±0.11c	65.9±1.08a	39.0±0.83b	18.0±0.49c	***	*n*.*s*.	*n*.*s*.
Total n-3 LC PUFA^9^	92.4±1.48c	110.1±1.77b	126.8±0.73a	87.5±1.88c	107.8±1.82b	127.0±2.27a	***	*n*.*s*.	*n*.*s*.
Total LC PUFA^10^	156.7±1.78a	149.0±1.36abc	144.5±0.82c	153.5±2.84ab	146.6±2.33b	145.0±2.65bc	***	*n*.*s*.	*n*.*s*.
*FA ratios*									
ARA/EPA ratio^11^	2.5±0.03b	0.9±0.03c	0.2±0.00d	2.7±0.03a	0.9±0.00b	0.2±0.01d	***	***	*n*.*s*.
LC PUFA n-3 /n-6^12^	1.4±0.02c	2.8±0.10b	7.2±0.03a	1.3±0.02c	2.8±0.03c	7.1±0.13a	***	*n*.*s*.	*n*.*s*.

Values in the same row with different superscripts are significantly different (P < 0.05) as determined by Student-Newman-Keuls post hoc multiple comparisons test. P values relative to two-way ANOVA statistical test are reported in the last three columns on the right hand side of the table. Further details relative to fatty acid digestibility and growth performances are avilable at Trullàs *et al*. (2015) and Norambuena *et al*. (2016).

^1^ n.s. = not significant

^2^ FA = fatty acids.

^3^ SFA = saturated fatty acids.

^4^ MUFA = monounsaturated fatty acids.

^5^ n-6 PUFA = omega-6 polyunsaturated fatty acids.

^6^ n-3 PUFA = omega-3 polyunsaturated fatty acids.

^7^ PUFA = polyunsaturated fatty acids.

^8^ n-6 LC-PUFA = long chain omega-6 polyunsaturated fatty acids.

^9^ n-3 LC-PUFA = long chain omega-3 polyunsaturated fatty acids.

^10^ Total long chain polyunsaturated fatty acid

^11^ARA/EPA = ratio of 20:4n-6/20:5n-3

^12^ LC-PUFA n-3/n-6 = ratio of long chain n-3 PUFA/n-6 PUFA.

* P<0.05

** P<0.01 and

*** P<0.001.

### Tissue RNA extraction and quantitative real-time PCR (qPCR)

Expression of 11 selected genes was studied by reverse transcription real time quantitative PCR (qPCR). One gene related to the release of LC-PUFA from phospholipids, cytolic calcium-dependent phospholipase A2 (c*pla2*). Five genes are related to fatty acid biosynthesis pathway, fatty acid synthase (*fas*), fatty acyl desaturases (*Δ5fad* and *Δ6fad*), and fatty acyl elongases (*elovl5a* and *elovl2*). Two genes are involved in liver fatty acid β-oxidation, including acyl-CoA oxidase (*aco*) and carnitine palmitoyl transferase-one (*cpt1*), for peroxisomal and mitochondrial β-oxidation, respectively. The remaining three genes are the transcription factors and major regulators of lipid metabolism, peroxisome proliferator-activated receptor-alpha (*pparα*), beta (*pparβ*) and gamma (*pparγ*), which in liver are suggested to be related to the regulation of genes involved in fatty acid homeostasis [65]. All primers used are reported in Table 3. For RNA extraction, samples were transferred into 2-ml screw-cap tubes containing 1 ml of TRIzol (Ambion, Life Technologies, Australia), with approximately 50 mg of 1 mm diameter zirconium glass beads, and were homogenized (TissueLyser LT, Quiagen, USA). Solvent extraction was performed following the manufacturer's instructions and RNA quality and quantity were assessed by gel electrophoresis (Bio-Rad Gel Doc XR Electrophoresis Unit, US) and spectrophotometry (NanoDrop ND-2000, Thermo Scientific, Wilmington, USA), respectively. Two micrograms of total RNA per sample were reverse transcribed into cDNA using the High-Capacity cDNA RT kit (Applied Biosystems, Life Technologies, USA), following the manufacturer's instructions, but using a mixture of random primers (1.5 μl as supplied) and anchored oligo-dT (0.5 μl at 400 ng/μl, Applied Biosystems, USA). Negative controls (containing no enzyme) were performed to check for genomic DNA contamination. A similar amount of cDNA was pooled from all samples and the remaining cDNA was diluted 50-fold with water. Quantification of the expression of three reference genes (Table 3) (elongation factor 1 alpha (*ef1a1*), beta actin (*bact*) and cofilin 2 (*cof2*), previously validated in studies with juvenile Atlantic salmon, was performed [37]. Amplifications were carried out in duplicate (Real-Time PCR Detection System, CFX96 Touch™ Bio-Rad, USA) in a final volume of 20 μl containing 5 μl (target genes) or 2 μl (reference genes) of diluted (1/50) cDNA, 0.5 μM of each primer and 10 μl SYBR GREEN qPCR Master Mix (SsoAdvanced™ Universal SYBR® Green Supermix, Bio-Rad, Australia) and included a systematic negative control (NTC-non template control). The qPCR profiles contained an initial activation step at 95°C for 2 min followed by 35 cycles of: 15 s of denaturation at 95°C, 15 s for annealing at the temperature indicated in Table 3 and then 15 s at 60 or 72°C for melting and 15 s at 95°C for dissociation. A melt curve was performed enabling confirmation of the amplification of a single product in each reaction. Non-occurrence of primer–dimer formation in the NTC was also confirmed. The amplification efficiency of the primer pairs was assessed by serial dilutions of the cDNA pool (Table 3), which also allowed conversion of threshold cycle (Cq) values to arbitrary copy numbers. Expression of the target genes is given as mean normalized values (± SD), corresponding to the ratio between copy numbers and a normalization factor determined for the average expression of the 3 reference genes using geNorm with a Stability value, M < 0.45 [66].

**Table 3:** qPCR primers.

Primer	Forward primer 5'-3'	Reverse primer 5'-3'	Accession no	Efficiency
*Target genes*				
*Δ5fad* ^1^	GTGAATGGGGATCCATAGCA	AAACGAACGGACAACCAGA	AF478472 ^16^	103
*Δ6fad* ^2^	CCCCAGACGTTTGTGTCAG	CCTGGATTGTTGCTTTGGAT	AY458652 ^16^	102
*elovl2* ^3^	CGGGTACAAAATGTGCTGGT	TCTGTTTGCCGATAGCCATT	TC91192 ^17^	100
*elovl5a* ^4^	ACAAGACAGGAATCTCTTTCAGATTAA	TCTGGGGTTACTGTGCTATAGTGTAC	AY170327 ^16^	101
*fas* ^5^	ACCGCCAAGCTCAGTGTGC	CAGGCCCCAAAGGAGTAGC	DW551395 ^16^	104
*Aco* ^6^	AAAGCCTTCACCACATGGAC	TAGGACACGATGCCACTCAG	TC145297 ^17^	101
*cpt1* ^7^	GTACCAGCCCCGATGCCTTCAT	TCTCTGTGCGACCCTCTCGGAA	AM230810 ^16^	100
*cpla2* ^8^	GTCGCTGGCTGGAGCTGTGG	AGCCCTATGGGCCCTGGTCA	NM_001141333 ^16^	102
*pparα* ^9^	TCCTGGTGGCCTACGGATC	CGTTGAATTTCATGGCGAACT	DQ294237 ^16^	100
*pparβ* ^10^	GAGACGGTCAGGGAGCTCAC	CCAGCAACCCGTCCTTGTT	AJ416953 ^16^	102
*pparγ* ^11^	CATTGTCAGCCTGTCCAGAC	TTGCAGCCCTCACAGACATG	AJ416951 ^16^	100
*srebp1* ^12^	TCTGGGGCGTTGGTGAGGTGTTAC	CAGGCTGGCAGTGTGAAGATTGAAGG	NM 001195819 ^16^	102
*Reference genes*				
*elf1α* ^13^	CTGCCCCTCCAGGACGTTTACAA	CACCGGGCATAGCCGATTCC	AF321836 ^16^	100
*βactin* ^14^	ACATCAAGGAGAAGCTGTGC	GACAACGGAACCTCTCGTTA	AF012125 ^16^	101
*cofilin2* ^15^	AGCCTATGACCAACCCACTG	TGTTCACAGCTCGTTTACCG	TC63899 ^17^	100

^1^Δ-5 fatty acyl desaturase.

^2^Δ-6 fatty acyl desaturase.

^3^Fatty acyl elongase 2.

^4^Fatty acyl elongase 5a.

^5^Fatty acid synthase.

^6^Acyl-CoA oxidase.

^7^Carnitine palmitoyl transferase 1.

^8^Cytosolic calcium-dependent phospholipase A2.

^9^Peroxisome proliferator-activated receptor alpha.

^10^Peroxisome proliferator-activated receptor beta.

^11^Peroxisome proliferator-activated receptor gamma.

^12^Sterol regulatory element binding protein 1.

^13^Elongation factor 1-alpha.

^14^Beta actin.

^15^Cofilin-2.

^16^GenBank (http://www.ncbi.nlm.nih.gov/).

^17^Atlantic salmon Gene Index (http://compbio.dfci.harvard.edu/tgi/).

### Statistical analysis

All data were reported as mean ± standard error (n = 3, for genes expression n = 6). Dataset comprised of data originated from the comparison of three dietary treatments and two environmental temperatures. The effects of diet, temperature and their interactions were assessed by two-way analysis of variance (ANOVA, n+36), following confirmation of normality and homogeneity of variance. The results of the two-way ANOVA and t-test are reported in tables and/or figures as: n.s. = not significant; * P<0.05; ** P<0.01 and *** P<0.001. Data were also subjected to a Student-Newman-Keuls post hoc multiple comparisons test for identifying homogeneous subsets. Correlation and Person’s correlation coefficient (R^2^) were calculated to explore correlations between *in vivo* fatty acid enzyme activities and their corresponding mRNA gene expression. All statistical analyses were computed using IBM SPSS statistic 20 (IBM Corporation, New York, USA).

## Results

### Summary of performance

As previously described the performance data relative to this experimentation have been published in separate articles [19, 64]. Briefly, dietary treatments had only minor, almost trivial, effects on fatty acid and nutrient digestibility, whereas water temperature resulted in altered digestibility values, with lower nutrient digestibility recorded at lower water temperature [19]. Mortality was minimal during the entire experimentation (<0.3%) and overall all performance of fish were optimal with food conversion ratio (FCR) varying from 1.0 to 1.1. At the end of the experimentation, the largest fish (273.1±6.99 g average weight) were those fed D-ARA/EPA and held at 20°C, and the smaller fish (212.8±5.58 g average weight) were those fed D-ARA and held at 10°C. One of the most important differences observed between fish held at the two different temperatures, and independently from dietary treatments, was in their feed intake. Fish at higher water temperature consumed significantly more (from 1.8 to 2.3 g/day per fish) feed than those at the lower temperature (from 1.2 to 1.4 g/day per fish) [64].

### Apparent *in vivo* FA β-oxidation

Statistically significant differences were observed in the apparent *in vivo* fatty acid β-oxidation (nmol/g/day) in juvenile Atlantic salmon fed the three experimental diets at the two temperatures (Table 4). Specifically, high water temperature led to a significant increase in total fatty acid β-oxidation (varying amongst dietary treatments from 1,425.8 to 1,645.3 nmol/g/day in fish held at 20°C and from 321.5 to 814.5 nmol/g/day in fish held at 10°C), and the same trend was also observed in the β-oxidation of total SFA, MUFA, in n-6 PUFA and n-3 PUFA classes. At both water temperatures, fish fed diets with higher ARA content showed greater ARA β-oxidation, with a similar pattern for n-6 LC-PUFA (D-ARA > D-ARA/EPA > D-EPA). Similarly, for D-EPA, a greater level of EPA and n-3 LC-PUFA apparent *in vivo* β-oxidation was detected (D-EPA> D-ARA-EPA > D-ARA). Independently of the dietary treatment, the fatty acid substrates more abundantly used for catabolism could be summarized as follow: MUFA > SFA > n-6 PUFA > n-3 PUFA; whilst the dietary supply of these fatty acid classes was MUFA > SFA > n-6 PUFA = n-3 PUFA. This clearly suggests that the β-oxidation of FA is directly proportional to their availability, but not for n-6 PUFA and n-3 PUFA, where n-6 PUFA appeared to be preferentially utilised over n-3 PUFA for catabolic processes. Observing the effects of the dietary treatments, it was shown that in fish held at both temperatures, the apparent *in vivo* β-oxidation of SFA and MUFA was lower in the group with highest dietary EPA (D-EPA) supply. The apparent *in vivo* β-oxidation of PUFA was lower in fish fed the diet containing both EPA and ARA (D-ARA/EPA), compared to the other two treatments.

**Table 4:** Apparent *in vivo* fatty acid β-oxidation (nmol/g/day; deduced by the whole body fatty acid balance method) in Atlantic salmon fed experimental diets with different ARA/EPA ratios and reared at two different water temperatures.

FA (nmol/g/day)^1^	10°C			20°C			*P* values^2^		
	ARA	ARA/EPA	EPA	ARA	ARA/EPA	EPA	Diet	Temp.	Inter.
14:0	1.3±0.8	11.4±6.0	*n*.*d*.^*8*^	19.5±1.4	19.5±8.8	19.8±1.8	*n*.*s*.	***	*n*.*s*.
16:0	105.0±27.2ab	40.0±15.8b	13.6±6.8b	229.3±11.9a	223.8±51.4a	226.4±31.8a	*n*.*s*.	***	*n*.*s*.
18:0	44.7±12.5ab	30.5±21.7ab	15±3.1b	82.6±1.3a	57.3±16.0ab	37.6±15.8ab	**	*	*n*.*s*.
20:0	6.3±0.7	4.3±2.2	0.5±0.5	7.0±3.8	2.5±1.5	1.9±0.9	*	*n*.*s*.	*n*.*s*.
22:0	9.5±0.9ab	7.9±0.4b	4.3±0.9b	15.8±0.3a	9.7±2.2ab	5.4±2.1b	***	*	*n*.*s*.
24:0	3.6±0.3ab	2.3±0.8ab	1.6±0.2b	6.2±0.2a	3.5±0.4ab	1.7±0.4ab	***	***	*
Total SFA^3^	170.3±41.2abc	96.5±33.3bc	19.9±6.3c	360.4±11.2a	316.3±73.2a	292.9±51.4ab	*n*.*s*.	***	*n*.*s*.
14:1n-5	1.7±0.1c	2.2±0.2bc	2.0±0.3c	3.3±0.2ab	3.7±0.4a	3.8±0.2a	*n*.*s*.	***	*n*.*s*.
16:1n-7	4.5±1.6ab	20.1±10.2ab	0.0±0.0b	36.1±3.5a	30.6±13.5ab	33.7±3.4ab	*n*.*s*.	***	*n*.*s*.
18:1n-7	9.1±4.1ab	13.3±8.1ab	3.5±2.0b	29.3±2.3a	27.8±8.0ab	26.7±2.1ab	*n*.*s*.	***	*n*.*s*.
18:1n-9	329.9±94.7a	326.8±174.4ab	56.7±28.3b	655.2±50.2a	600.4±134.6a	541.3±58.6b	**.	***	*n*.*s*.
20:1n-9	0.2±0.2	2.4±2.4	*n*.*d*.	*n*.*d*.	0.2±0.2	*n*.*d*.	*n*.*c*.^*9*^	*n*.*c*.	*n*.*c*.
20:1n-11	1.6±0.0ab	1.5±0.1ab	0.4±0.2	2.5±0.0a	1.9±0.0b	1.3±0.0	***	***	*n*.*s*.
22:1n-11	1.6±0.4	1.6±0.7	*n*.*d*.	2.5±0.2	1.2±0.5	1.3±0.4	*n*.*s*.	*n*.*s*.	*n*.*s*.
22:1n-13	0.8±0.0e	1.7±0.2c	2.5±0.0b	1.1±0.0d	2.2±0.0b	3.7±0.0a	***	***	***
Total MUFA^4^	349.4±99.6ab	369.6±191.2ab	57.3±10.7b	730.8±56.1a	676.0±156.3a	611.8±58.2a	*n*.*s*.	***	*n*.*s*.
18:2n-6	113.9±21.7abc	39.6±8.6c	81.5±3.3bc	200.0±10.4a	178.5±34.9a	169.4±18.1ab	*n*.*s*.	***	*n*.*s*.
20:4n-6	63.5±15.9b	4.8±0.4c	1.4±0.7c	103.7±7.6a	48.4±1.7b	1.8±0.5c	***	***	*n*.*s*.
Total n-6 PUFA^5^	180.7±40.7bc	48.2±8.8d	83.7±4.5cd	308.4±18.5a	228.7±34.9ab	178.2±18.3bc	***	***	*n*.*s*.
18:3n-3	114.1±13.5bc	46.1±11.2c	127.1±4.0b	227.7±7.4a	200.5±22.6a	204.3±19.5a	**	***	*n*.*s*.
20:5n-3	*n*.*d*.	46.7±1.7bc	50.4±1.3bc	17.9±3.3c	88.3±10.6b	138.6±9.5a	***	***	**
22:6n-3	*n*.*d*.	*n*.*d*.	*n*.*d*.	*n*.*d*.	*n*.*d*.	*n*.*d*.	*n*.*c*.	*n*.*c*	*n*.*c*
Total n-3 PUFA^6^	114.1±13.5d	77.2±10.8d	160.5±13.8cd	245.6±7.5bc	288.7±32.5ab	342.9±27.9a	*n*.*s*.	***	*n*.*s*.
Total PUFA^7^	294.9±49.9b	125.4±14.0b	244.2±15.7b	554.1±25.5a	517.4±66.8a	521.1±46.1a	***	***	n.s.
*Total Oxidation*	814.5±189.0b	591.5±225.3b	321.5±11.6b	1,645.3±90.0a	1,509.7±296.0a	1,425.8±155.4ab	n.s.	***	n.s.

^1^ apparent *in vivo* fatty acid β-oxidation (nmol/g/day).

^2^ FA = fatty acids.

^3^ SFA = saturated fatty acids.

^4^ MUFA = monounsaturated fatty acids.

^5^ n-6 PUFA = omega-6 polyunsaturated fatty acids.

^6^ n-3 PUFA = omega-3 polyunsaturated fatty acids.

^7^ PUFA = polyunsaturated fatty acids.

^8^ not detected.

^9^ not computed.

### Apparent *in vivo* fatty acid bioconversion

Statistically significant differences were observed in the apparent *in vivo* fatty acid bioconversion in juvenile Atlantic salmon fed the three experimental diets at the two temperatures (Table 5). Specifically, high water temperature (20°C) caused a significant reduction in *Δ6fad* and *elovl2*, whereas *elovl5a* apparent *in vivo* activity was not affected by water temperature. Diets with higher ARA content showed greater bioconversion of ARA into longer and more unsaturated n-6 LC PUFA at both temperatures (10°C and 20°C). Similarly, for EPA, a greater level of EPA bioconversion toward longer and more unsaturated n-3 LC-PUFA was found with increasing dietary supply. However, no differences in the final DHA biosynthesis (elongation of 22:5n-3 to 24:5n-3, Δ6-desaturation of 24:5n-3 to 24:6n-3 and peroxisomal chain shortening of 24:6n-3 to 22:6n-3) were observed, in response to dietary treatment or water temperature. Interestingly, the only group with detectable ARA-biosynthesis from its precursor 20:3n-6 was the D-EPA (low in ARA) treatment for fish held at 20°C. Inversely, the only two groups with detectable EPA-biosynthesis from its precursor 20:4n-3, were the D-ARA and D-ARA/EPA treatments for fish held at 10°C. In fish held at lower temperature (10°C), ARA-biosynthesis was not detected, and in fish held at higher temperatures (20°C), EPA-biosynthesis was not detected.

**Table 5:** Apparent *in vivo* activity (nmol/g/day) of the key enzymes in fatty acid biosynthetic pathways (deduced by the whole body fatty acid balance method) in Atlantic salmon fed experimental diets with different ARA/EPA ratios and reared at two different water temperatures.

FA (nmol/g/day)^2^	10°C			20°C			*P* values		
	ARA	ARA/EPA	EPA	ARA	ARA/EPA	EPA	Diet	Temp.	Inter.
*Neogenesis*									
12:0	5.6±2.6	4.1±2.8	4.3±2.3	2.0±0.1	2.6±0.6	3.3±0.7	*n*.*s*.	*n*.*s*.	*n*.*s*.
*D9fad*	n.d.	0.6±0.1b	4.9±0.6a	n.d.	n.d.	n.d.	***	*n*.*c*.	*n*.*c*.
16:0 to 16:1n-7	n.d.	0.6±0.1b	4.6±0.2a	n.d.	n.d.	n.d.	***	*n*.*c*.	*n*.*c*.
18:0 to 18:1n-9	n.d.	n.d.	n.d.	n.d.	n.d.	n.d.	*n*.*c*.	*n*.*c*.	*n*.*c*.
*D6fad*	144.6±3.8	134.5±33.6	110.9±2.4	86.6±8.4	87.8±19.6	115.0±8.5	**	**	*n*.*s*.
18:2n-6 to 18:3n-6	3.5±1.0bc	8.4±0.4bc	11.7±0.8ab	0.0±0.0c	3.0±1.1c	18.8±4.0a	***	*n*.*s*.	*n*.*s*.
24:4n-6 to 24:5n-6	11.5±1.5a	5.5±0.9ab	1.9±0.6b	13.0±1.6a	10.3±1.6a	5.3±2.2ab	***	*	*n*.*s*.
18:3n-3 to 18:4n-3	71.3±4.7a	45.1±17.7ab	24.5±1.1b	32.3±2.6ab	19.8±8.2b	19.7±1.8b	*	**	*n*.*s*.
24:5n-3 to 24:6n-3	58.3±4.8	75.5±14.7	72.2±3.4	41.3±7.1	54.8±13.5	71.2±12.3	*n*.*s*.	*n*.*s*.	*n*.*s*.
*D5fad*	31.3±5.7	22.4±2.3	n.d.	n.d.	n.d.	12.1±1.5	*n*.*c*.	*n*.*c*.	*n*.*c*.
20:3n-6 to 20:4n-6	n.d.	n.d.	n.d.	n.d.	n.d.	12.1±1.5	*n*.*c*.	*n*.*c*.	*n*.*c*.
20:4n-3 to 20:5n-3	31.3±5.7	22.4±2.3	n.d.	n.d.	n.d.	n.d.	*n*.*c*.	*n*.*c*.	*n*.*c*.
*Elovl5 & Elovl2*	254.0±18.5ab	284.5±49.2ab	301.1±18.9ab	155.7±23.0b	247.2±59.3ab	341.8±13.1a	**	*n*.*s*.	*n*.*s*.
12:0 to 14:0	0.1±0.1	2.0±2.0	5.3±2.4	n.d.	n.d.	n.d.	*n*.*s*.	*n*.*s*.	*n*.*s*.
14:0 to 16:0	n.d.	n.d.	n.d.	n.d.	n.d.	n.d.	*n*.*c*.	*n*.*c*.	*n*.*c*.
16:0 to 18:0	n.d.	3.2±3.2	7.5±0.9	n.d.	n.d.	n.d.	*n*.*s*.	*n*.*c*.	*n*.*c*
18:1n-9 to 20:1n-9	n.d.	n.d.	n.d.	n.d.	n.d.	n.d.	*n*.*c*.	*n*.*c*.	*n*.*c*.
18:2n-6 to 20:2n-6	13.5±1.5	8.4±4.2	10.4±1.0	12.3±1.9	12.8±1.5	11.2±4.7	*n*.*s*.	*n*.*s*.	*n*.*s*.
18:3n-6 to 20:3n-6	7.4±0.3ab	4.7±2.2ab	8.5±1.3ab	1.0±0.5b	3.6±1.2ab	12.3±4.3a	*	*n*.*s*.	*n*.*s*.
20:4n-6 to 22:4n-6	20.4±2.8a	10.2±2.4ab	1.8±0.8b	20.6±2.6a	14.7±2.0a	2.7±0.5b	***	*n*.*s*.	*n*.*s*.
22:4n-6 to 24:4n-6	11.5±1.5a	5.5±0.9ab	1.9±0.6b	13.0±1.6a	10.3±1.6a	5.3±2.2ab	***	*	*n*.*s*.
18:3n-3 to 20:3n-3	2.6±0.6b	6.4±3.0b	18.6±0.6a	1.5±0.8b	25.1±2.6a	27.0±1.9a	***	**	**
20:3n-3 to 22:3n-3	3.7±0.5c	5.7±0.5bc	7.6±0.5b	4.0±0.3c	6.7±0.4b	9.7±0.4a	***	**	*n*.*s*.
18:4n-3 to 20:4n-3	37.2±6.3a	33.9±13.3ab	8.9±0.9ab	4.2±1.0b	8.1±2.4ab	10.2±1.1ab	*n*.*s*.	**	*n*.*s*.
20:5n-3 to 22:5n-3	73.2±3.8ab	108.3±5.8ab	129.4±20.6a	44.6±14.9b	84.5±26.3ab	144.1±5.2a	**	*n*.*s*.	*n*.*s*.
22:5n-3 to 24:5n-3	60.8±5.2	76.4±15.2	73.6±4.2	41.8±9.8	64.4±22.8	94.2±11.4	*n*.*s*.	*n*.*s*.	*n*.*s*.
*Elovl5a*	33.3±5.9	30.6±2.8	35.0±5.0	31.1±2.9	51.9±12.2	66.8±5.5	*n*.*s*.	*n*.*s*.	*n*.*s*.
*Elovl2*	94.8±2.6a	99.2±20.1a	100.4±6.2a	59.0±14.9b	60.1±8.8b	90.5±0.5a	*	*	*n*.*s*.
*Peroxisomal chains shortening*									
	55.9±1.5	67.8±10.4	64.3±4.5	43.7±6.5	62.2±20.1	83.4±14.4	*n*.*s*.	*n*.*s*.	*n*.*s*.

^1^Δ-5 fatty acyl desaturase.

^2^Δ-6 fatty acyl desaturase.

^3^ Stearoyl-CoA 9-desaturase.

^4^ Δ6 desaturase.

^5^ Δ5 desaturase.

^6^ Sum of elongase 5 and 2.

^7^ elongase 5a.

^8^ elongase 2.

^9^Peroxisome proliferator-activated receptor alpha.

^10^Peroxisome proliferator-activated receptor beta.

### ARA and EPA metabolic fate

**Fig 1:**
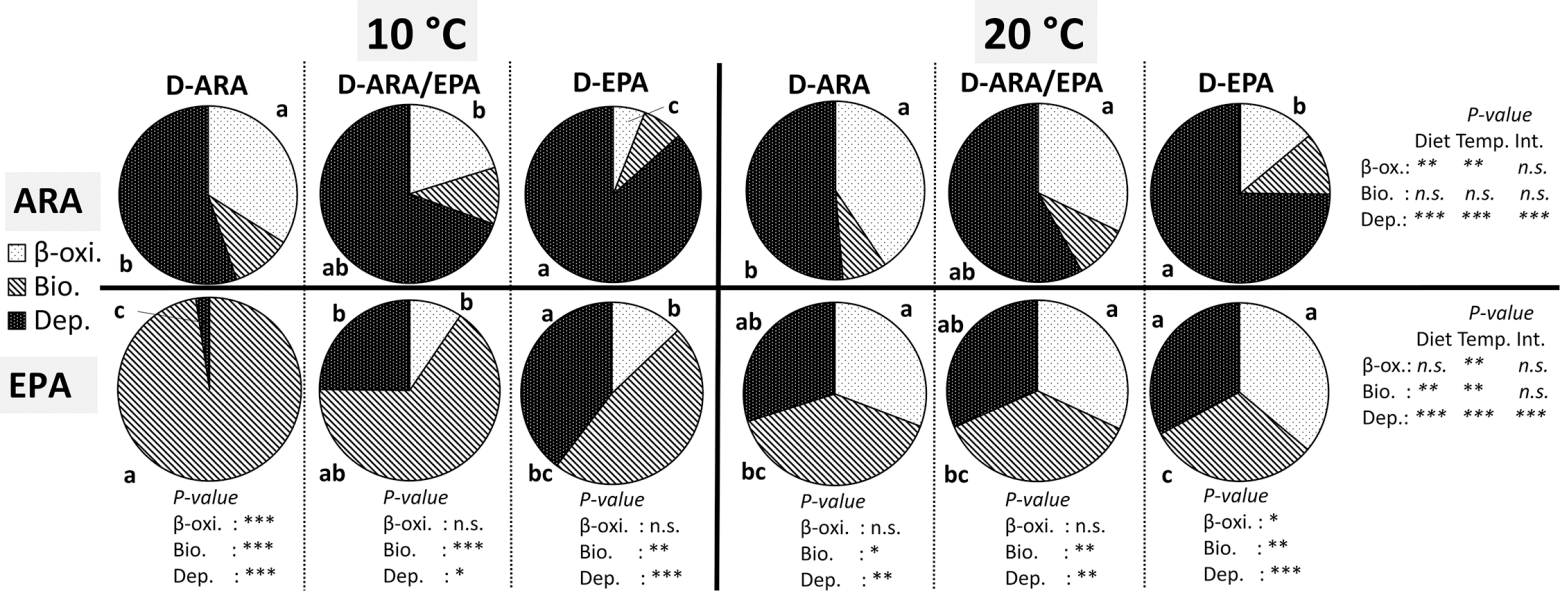
Metabolic fate of dietary ARA and EPA (β-oxidation, bioconversion and deposition, expressed as % of net intake) in juvenile Atlantic salmon fed experimental diets with different ARA/EPA ratios and reared at two different water temperatures. For ARA and EPA fate, from left to right, values in the same row, and for the same category (β-oxi., Bio. and Dep.), with different superscripts are significantly different (P < 0.05) and *P* values relative to the two-way ANOVA are reported on the right (n.s. = not significant; * P<0.05; ** P<0.01 and *** P<0.001). Statistical results (P-values) of the T- Student test is reported at the bottom of each column, comparing EPA and ARA for each dietary treatment and for the same category (β-oxi., Bio. and Dep.). Legend: i) Experimental diets abbreviations: D-ARA (EPA/ARA ratio = 0.5), D-ARA/EPA (EPA/ARA ratio = 1.5) and D-EPA (EPA/ARA ratio = 9); ii) 10°C and 20°C refer to the water temperature in which juvenile salmon were reared on; iii) β-oxi. = β-oxidation; Bio. = Bioconversion; and Dep. = Deposition.

The metabolic fate (expressed as % of the net intake) of ARA and EPA towards β-oxidation, bioconversion or direct deposition in Atlantic salmon was significantly affected by water temperatures (10°C and 20°C) and by the dietary treatments (dietary ARA/EPA) (Fig 1).

As for ARA, the main metabolic fate was deposition, which was significantly higher in D-EPA group (up to 84%) compared to the other two dietary treatments, and was also affected by temperature (higher deposition at lower temperature). The second quantitatively most abundant fate of dietary ARA was β-oxidation, which was significantly affected by diet and also temperature, with higher β-oxidation in fish receiving higher dietary supply of ARA, and, for the same dietary supply, higher β-oxidation in fish held at higher temperature. ARA-bioconversion to longer and more unsaturated FA was not affected by either temperature or diet, being about 10% of net dietary intake.

Rather different trends were observed for the metabolic fate of dietary EPA. Overall, the main metabolic fate was bioconversion towards longer and more unsaturated FA, followed by deposition and then β-oxidation. In fish held at the higher water temperature (20°C) the metabolic fate of dietary EPA was practically equally split across the three possible pathways (about 30% each) and was not affected by the dietary supply of EPA. On the contrary, in fish at 10°C, marked effects of the dietary treatment were observed, with bioconversion being highest in fish receiving limited dietary EPA (D-ARA), and being reduced with the increase of dietary EPA availability, whilst deposition and β-oxidation increased proportionally with the increase of dietary EPA availability (Fig 1).

### Genes expression levels

**Fig 2:**
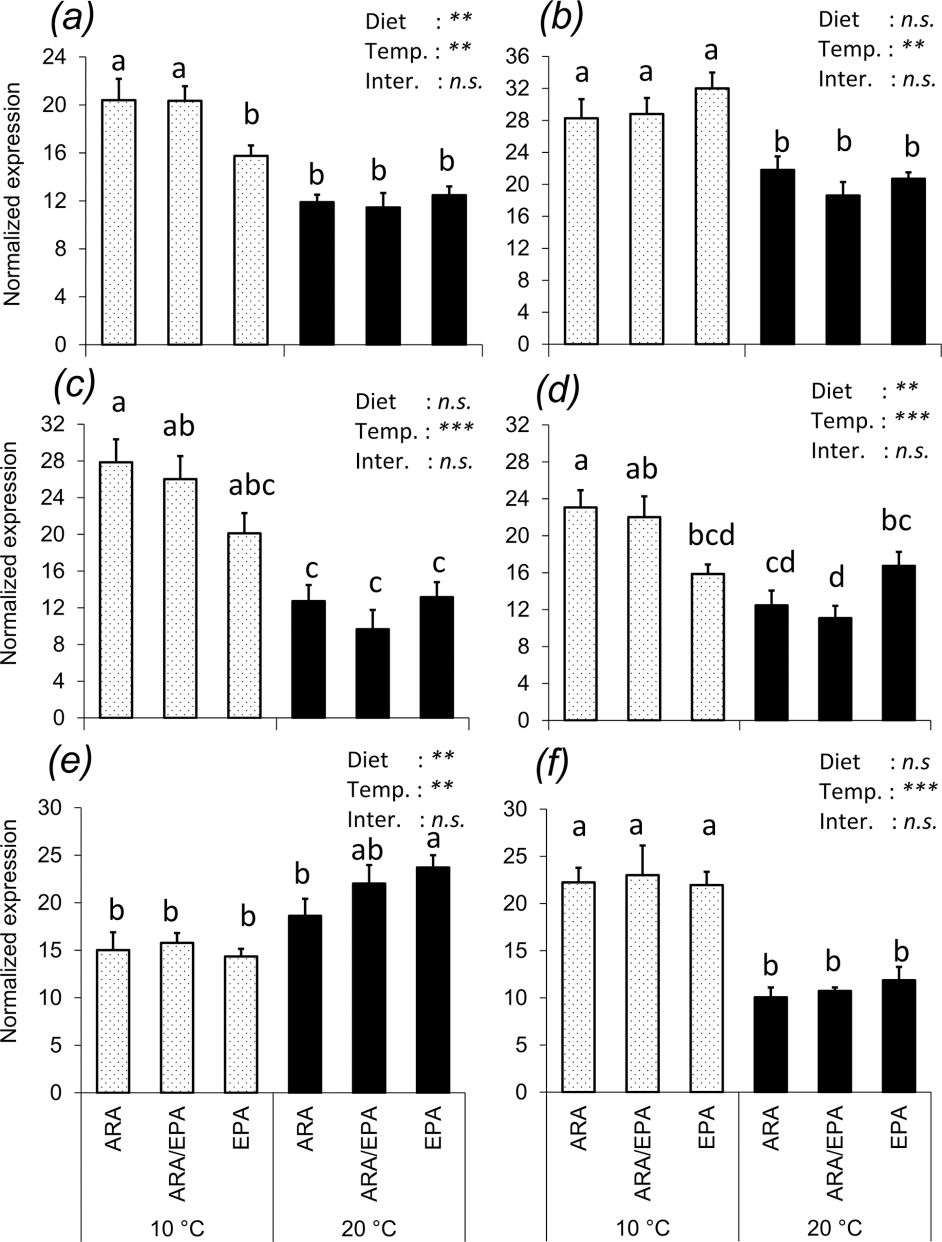
Hepatic expression of genes related to fatty acid biosynthesis and metabolism in juvenile Atlantic salmon fed diets containing different ARA/EPA levels and reared at two different temperatures. Results are normalized expression values of (A) fatty acid synthase (*fas*), (B) cytosolic calcium-dependent phospholipase A2 (c*pla2*), (C) Δ6 fatty acyl desaturase (Δ*6fad*), (d) Δ5 fatty acyl desaturase (Δ*5fad*), (e) fatty acyl elongase 5a (*elovl5a*) and (f) fatty acyl elongase 2 (*elovl2*). Normalized values are treatment means of n = 6, with standard errors represented by vertical bars. Mean values with unlike letters are statistically different between treatments (P<0.05, ANOVA).

**Fig 3:**
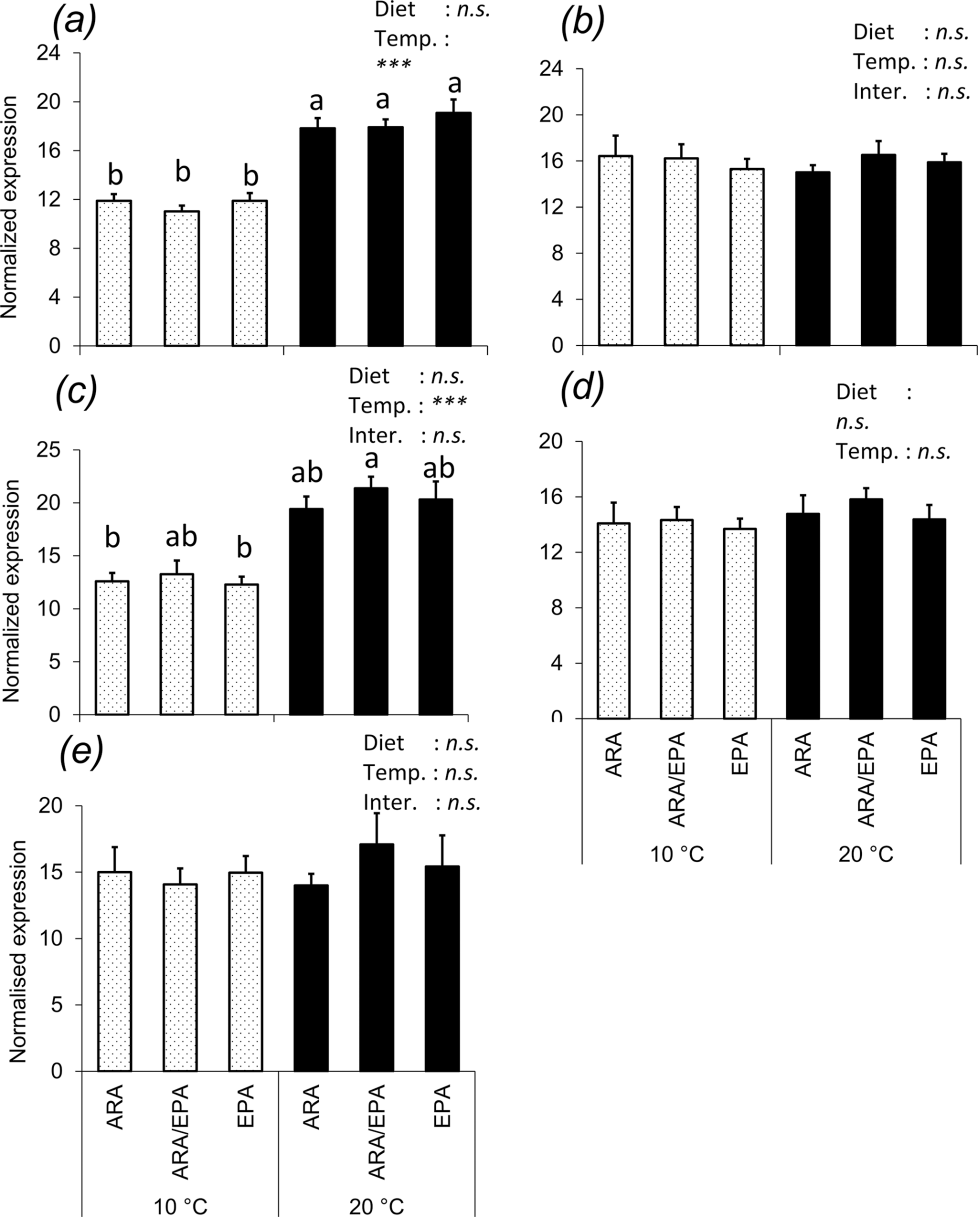
Hepatic expression of genes related to fatty acid β-oxidation and regulation of lipid metabolism in juvenile Atlantic salmon fed diets containing different ARA/EPA levels and reared at two different temperatures. Results are normalized expression values of (a) carnitine palmitoyl transferase-1 (*cpt1*), (b) acyl-CoA oxidase (*aco*) and (c) peroxisome proliferator-activated receptor alpha (*pparα*), (d), peroxisome proliferator-activated receptor beta (*pparβ*) and (e), peroxisome proliferator-activated receptor gamma (*pparγ*). Normalized values are treatment means of n = 6, with standard errors represented by vertical bars. Mean values with unlike letters are statistically different between treatments (P<0.05, ANOVA).

Statistically significant differences were found in the expression of several key genes for metabolic pathways relative to fatty acid (and their derivatives) biosynthesis, β-oxidation and their regulation in juvenile Atlantic salmon fed different experimental diets at two water temperatures (Figs 2 and 3). The expression of genes related to fatty acid biosynthesis, in particular, *de novo* synthesis (*fas*), phospholipids’ fatty acid release (c*pla2*) and fatty acyl desaturation (*Δ5fad* and *Δ6fad*) and elongation (*elovl2*) were significantly reduced with increase in temperature, whilst the opposite was observed for *elovl5a* expression (Fig 2).

Regarding the effects of diets, mRNA levels of *Δ5fad* were significantly increased in D-EPA at the higher temperature, whereas at the lower temperature *Δ5fad* expression was increased in D-ARA. A similar trend was also observed for the expression of *Δ6fad*, even if not statistically significant for those fish at the higher water temperature. In fish at higher water temperature, a dietary effect was also observed on e*lovl5a* transcription, being up-regulated in D-EPA compared to D-ARA.

In the case of genes related to fatty acid β-oxidation and of transcription factors involved in the regulation of lipid metabolism, a significant effect of temperature, but not diet, was apparent in *cpt1* and ppar*α*, which had decreased mRNA levels in the groups of fish held at low temperature (10°C). On the other hand, *aco*, *pparβ* and *pparγ* were regulated neither by dietary treatments nor temperature (Fig 3).

**Fig 4:**
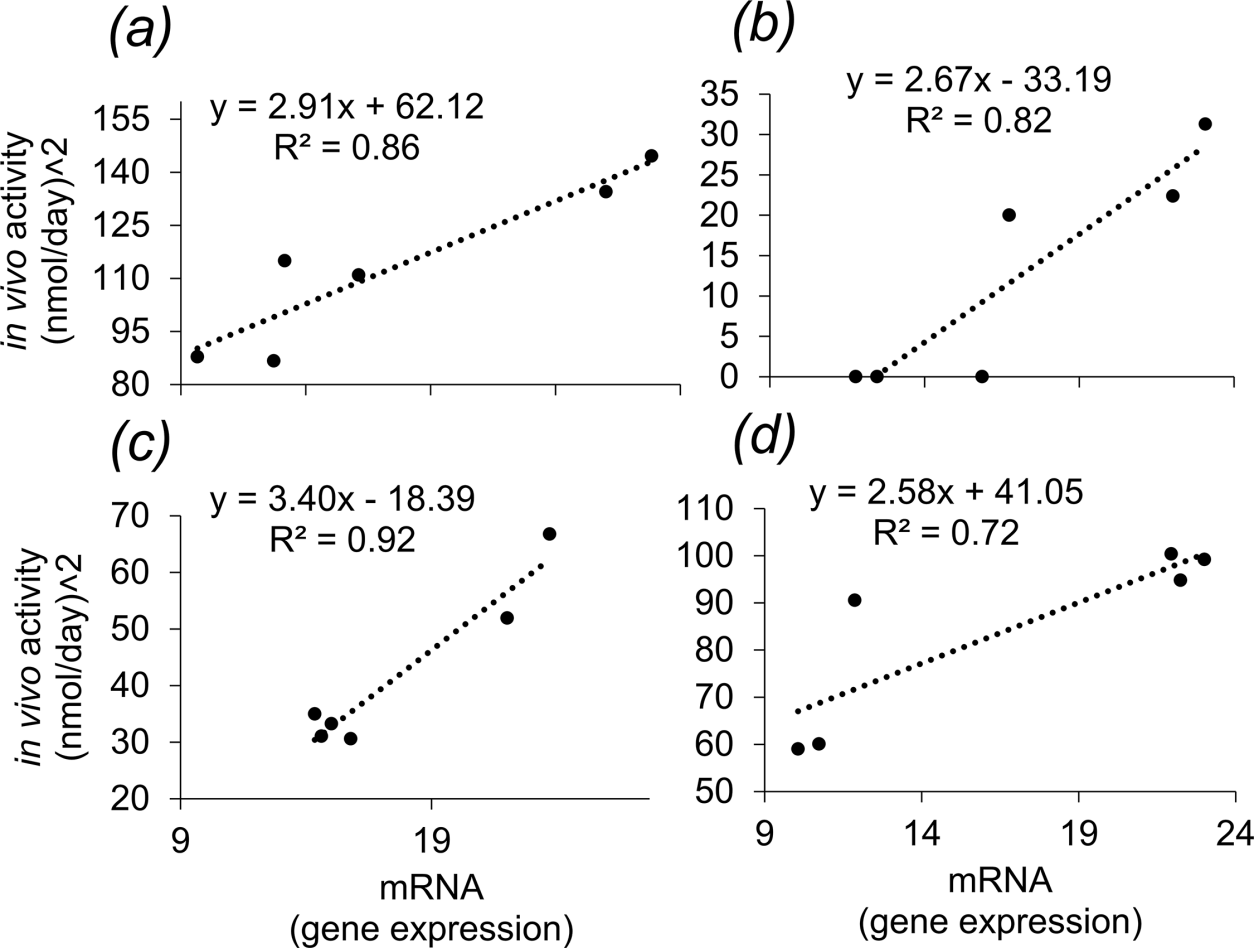
Correlation and linear regression between mRNA levels (X) and apparent *in vivo* enzyme activity (Y) for (a) Δ6 fatty acyl desaturase (Δ*6fad*), (b) Δ5 fatty acyl desaturase (Δ*5fad*), (c) fatty acyl elongase 5a (*elovl5a*), and (d) fatty acyl elongase 2 (*elovl2*) (R^2^ = Person’s correlation coefficient).

Significant linear relationships were found between the mRNA levels and the apparent *in vivo* enzyme activity of desaturases and elongases (Fig 4). The Person’s correlation coefficients (R^2^) for *Δ6fad*, *Δ5fad*, *elov5a* and *elov2* were 0.86, 0.82, 0.92 and 0.72 respectively.

## Discussion

This study reports a series of novel findings relative to the roles of dietary ARA and EPA and how their metabolic utilisation, and likely also their dietary requirements for optimal performance, can be affected by changes in their relative levels and by water temperature in Atlantic salmon.

A first and clear finding of this study was that the water temperature, independently of dietary treatment, had a significant effect on the β-oxidation of a wide range of FA, as indicated by the increased apparent *in vivo* enzyme activity and expression of *cpt1* and *pparα* in fish held at the higher water temperature. In addition to the normal, and expected, increase in basal metabolism resulting from the higher body temperature, it has been suggested that with an increase in environmental temperature fish increase swimming activity [26], which in turn should directly increase energy consumption. A more recent study on the fish’s capacity to adapt to high water temperatures showed the up regulation of several genes involved in a variety of metabolic processes, suggesting a shift in energy production for maintaining performance at elevated temperatures, which is envisaged as critical for improved aerobic scope and fish resilience [28]. Additionally, a study on the energy used by rainbow trout (*Onchorynchus mykiss*) during swimming at high temperatures, suggested that when fish are expose to higher temperature (from 5°C to 15°C), lipid remained the most important fuel source contributing up to 55% of total energy, with an increase also in the relative energy contribution from carbohydrate (from 15% to 25%) [26]. Furthermore, several studies showed that an increase in water temperature induced an increased lipid β-oxidation primarily in red muscle, but also in white muscle of teleost fish [24, 67, 68].

On the contrary, other studies showed that the increase in water temperature was responsible for reduced β-oxidation in hepatocytes and intestinal enterocytes in teleost fish [25, 69]. It is important at this point to draw attention to the tissues analysed in these studies: liver and intestine showed a reduction of fatty acid β-oxidation with increased temperature, whilst muscle showed an increase of fatty acid β-oxidation with increased temperature. Independently of the catabolic activity of each tissue, given the contribution of muscle to the overall animal body weight, it appears evident that the total fatty acid β-oxidation activity of a fish will be primarily determined by the muscle [70], which is in agreement with the findings of the present study, utilising a whole body approach. Additionally, in liver the peroxisomal β-oxidation is the primary route for fatty acid catabolism, whilst in muscle β-oxidation occurs principally in the mitochondria [70]. Concurrently, this effect of water temperature on β-oxidation was also reflected in the gene expression results. With regard to mitochondrial β-oxidation, the expression of *pparα*, a transcription factor regulating the expression of several lipid metabolism, including β-oxidation, genes [33, 71] and that of *cpt1*, a key enzyme responsible for the movement of long-chain FA from the cytosol into the mitochondria for β-oxidation, were both increased at the higher water temperature. On the other hand, hepatic mRNA levels of *aco*, a rate-limiting enzyme in the peroxisomal β-oxidation, were not modulated by water temperature. Therefore, these findings suggest that high water temperatures in juvenile Atlantic salmon increased fatty acid β-oxidation in the mitochondria, rather than in peroxisomes, and that the whole body increase in β-oxidation probably reflects changes occurring more in muscle than in liver tissues [71, 72]. Similarly, a previous study in Atlantic salmon also suggested that water temperature mainly regulates mitochondrial β-oxidation [25], which can be up to 6-fold higher than peroxisomal oxidation [67], hence determining whole-body total fatty acid catabolic processes. Eventually, most of the energy produced by β-oxidation should promote growth, which is in agreement with, and reflected by, the greater performance of fish held at higher water temperature, as previously reported by Norambuena *et al*. (2016). In line with this, the mRNA level of genes involved in pathways associated to fish growth and tissue development has been suggested as a good indicator of a fish’s capacity to adapt to sup-optimal temperatures [28]. Therefore, a practical application of these results is that it appears advisable to formulate and utilise diets with higher energy content for fish reared at elevated water temperatures.

It has been documented that an increase in fish growth rate boosts the catabolism of LC-PUFA, when these are provided in surplus [23]. Observing the *in vivo* metabolic fate of dietary EPA and ARA recorded in the present study, it was evident that LC-PUFA β-oxidation was mediated by water temperature and growth, but also by their dietary content, particularly for EPA. In this case, EPA was more catabolised when fish were fed the EPA and ARA/EPA diets, and there was also a significantly increased β-oxidation in fish held at 20°C. This may suggest that EPA overall requirements are reduced at elevated temperatures. A previous study on the effects of water temperature on fatty acid metabolism in freshwater fish (*Cyprinus carpio*) showed that the deposition of radio-labelled EPA was affected by temperature, with a strong reduction of its deposition and retention in tissues of fish held at high temperature (25°C) suggesting a modification on membrane cell an increased β-oxidation of this fatty acid [14, 22]. Accordingly, this increased catabolism of EPA may advocate that dietary EPA requirement for optimal performance could be somewhat reduced in fish held at elevated water temperature, as also previously suggested [64]. Observing the metabolic fate of dietary EPA (as % of net intake towards deposition, β-oxidation or bioconversion) at higher water temperature, there was no effect of the dietary treatment despite the large differences in its dietary supply. Therefore, this may suggest that dietary EPA is somewhat of little importance at higher water temperature and that as little as the amount provided by D-ARA diet is sufficient to fulfil basic requirements, given that there was no metabolic attempt to preserve it from catabolism.

Alternatively, observing the metabolic fate of dietary ARA, it appeared that ARA was primarily deposited, independently of water temperature, and clear effects of dietary treatments were also evident, with increased ARA deposition (as % of net intake) when ARA availability was reduced. This suggests the existence of a metabolic attempt towards preserving ARA at both studied water temperatures. In a previous study it was reported that ARA accumulation in juvenile Atlantic salmon occurred primarily in the liver and that it increased significantly over time [64]. A study on broodstock Senegalese sole (*Solea senegalensis*) showed that fish can modulate their ARA required relative to water temperature and if dietary ARA is not provided in adequate supply, male fish quite efficiently biosynthesise it from its precursor [42]. However these activities can be specie- and sex- dependent conditioned by several factors including water temperature and fish ontogeny.

Competition between ARA and EPA occurs at several points in their metabolism. Firstly, their synthesis from their respective n-6/n-3 precursors occurs through the same enzymatic pathway; secondly, ARA and EPA compete for the same enzymes catalysing their incorporation in membrane phospholipids; and thirdly both FA are metabolized by the same enzymes in the eicosanoid production pathway [32], which could be a crucial aspect of the fish’s physiological acclimation capacity to sub-optimal water temperatures [28, 33]. Previous studies have shown that low-activity anti-inflammatory eicosanoids produced by EPA enzymatic oxidation can be drastically reduced with low dietary EPA intake [7, 55, 58]. Similarly, high levels of EPA reduce the secretion of high activity pro-inflammatory eicosanoids produced by ARA-enzymatic oxidation, which are necessary for an appropriate inflammatory response [73]. It is well know that the inflammatory response is regulated by eicosanoids, and contrary to modern-, western societies-, human diets containing high levels of ARA and low level of EPA and being considered excessively pro-inflammatory [74], the level of EPA in fish could be too high, relative to dietary ARA availability, with a possible detrimental effects on the fish’s resilience and adaptability to sub-optimal environmental conditions, where a “healthy” and adequate inflammatory response is needed. Eicosanoid regulation at high water temperature has not been considered here and should be elucidated in further studies.

From an anabolic point of view, the results obtained in this study showed that at lower water temperature there was an up regulation of hepatic mRNA’s coding for genes involved in fatty acid *de novo* formation (*fas*), as well as of those involved in EPA and ARA membrane mobilization and bioconversion (c*pla2*), and fatty acyl desaturation (*Δ6fad* and *Δ5fad*) and elongation (*elovl2*). These results are consistent with a well-documented notion that fatty acid bioconversion toward longer and more unsaturated LC-PUFA is promoted in fish held at low water temperatures [69, 75–77]. However, an opposite trend was observed for *elovl5a*, which is much less responsive to changes in dietary LC-PUFA levels than other enzymes (desaturases and *elovl2*) of the LC-PUFA biosynthesis pathway [78]. Furthermore, fatty acyl elongates interact with reductases and dehydratase and it can also act on different pathways [79] and therefore this mRNA response might reflect these interaction or changes in other pathways, rather than in LC-PUFA biosynthesis.

A highly interesting trend was observed in mRNA levels of *Δ5fad* with respect to dietary treatments, which was also directly and linearly correlated with the apparent *in vivo* enzyme activity recorded. At lower temperature, the highest *Δ5fad* expression was recorded in fish with limited dietary EPA and abundant ARA supply (D-ARA), whilst at higher temperature, the highest mRNA level was recorded in fish with abundant dietary EPA and limited ARA supply (D-EPA). Furthermore, a similar trend was also observed for *Δ6fad* activity and gene expression. To understand the possible physiological explanation for this trend, it is important to keep in mind the dietary supply of EPA and ARA, and that these enzymes are those responsible for the actual biosynthesis of EPA and ARA. Thus, at lower temperature, increased transcription and activity of the enzymes is triggered by low dietary EPA, suggesting that fish metabolism is attempting to increase total EPA availability as a possible cellular homeostatic response [14]. At higher temperature, on the other hand, increased transcription and activity of these enzymes is triggered by low dietary ARA, therefore suggesting that fish metabolism is attempting to increase total ARA availability. These observations, coupled with those reported above relative to EPA β-oxidation, clearly suggest that at higher water temperature fish might lower their apparent requirements for optimal performance of EPA and, conversely, require more ARA.

Considering that evolution and adaptation might have potentially similar effects in inducing metabolic changes, in agreement with the finding of this study, warm water finfish species have been suggested to display higher elongase and desaturase activities toward the biosynthesis of n-6 LC-PUFA compared to the cooler water species [80]. In addition, a study with wild caught fish showed elevated levels of ARA in tissues of fish living in warmer waters [81]. A recent study investigating the fish capacity to acclimatise to warmer temperatures, showed that both immune- and stress- responsive genes were up-regulated in fish held at high temperatures, indicating a clear basal inflammatory response, modulated by high water temperatures, allowing fish to better cope with this sub optimal environmental conditions [28]. ARA is a precursor of highly bioactive pro-inflammatory eicosanoids, which are involved not just in immune responses, but also in cardiovascular physiology by mediating different biological processes, including blood flow and vasodilatation [51, 52]. Cardiovascular failures have been reported when fish are held at high water temperatures, [82, 83] and are fed commercial diets which typically contain limited levels of ARA. These results and evidences clearly point toward the fact that ARA plays a very important role in fish held at sub-optimal elevated water temperatures.

In conclusion, the present study clearly supports the hypothesis that dietary ARA/EPA requirements for optimal performance in Atlantic salmon are affected by water temperature, which in turn might be adjusted by fish’s acclimation and physiological response in sub-optimal water temperature. In practical terms, results presented here suggest that diets for Atlantic salmon maintained at sub-optimal high water temperatures should have higher energetic content, and relatively lower content of EPA and, conversely, a higher ARA content, for “priming” and maintaining a basal pro-inflammatory response.

This is a novel and fundamental information that warrants industry and scientific attention, in consideration of the imminent increase in water temperatures, continuous expansion of aquaculture operations, resources utilisation in aquafeed and much needed seasonal/adaptive nutritional strategies towards achieving increased economic and environmental sustainability of the aquaculture sector.
